# Missed opportunities to improve food security for pregnant people: a qualitative study of prenatal care settings in Northern New England during the COVID-19 pandemic

**DOI:** 10.1186/s40795-022-00499-7

**Published:** 2022-01-24

**Authors:** Chelsey R. Canavan, Tiffany D’cruze, Meaghan A. Kennedy, Kayla E. Hatchell, Maureen Boardman, Arvind Suresh, Daisy Goodman, Alka Dev

**Affiliations:** 1 Department of Population Health, Dartmouth-Hitchcock Medical Center, Medical Center Drive, Lebanon, NH 03766 USA; 2 The Dartmouth Institute for Health Policy and Clinical Practice, Geisel School of Medicine at Dartmouth, Hanover, NH USA; 3 Department of Community and Family Medicine, Geisel School of Medicine at Dartmouth, Hanover, NH USA; 4 Department of Obstetrics and Gynecology, Dartmouth-Hitchcock Medical Center, Lebanon, NH USA

**Keywords:** Food security, Nutrition, Maternal health, Social determinants of health, Prenatal care

## Abstract

**Background:**

Food insecurity during pregnancy has important implications for maternal and newborn health. There is increasing commitment to screening for social needs within health care settings. However, little is known about current screening processes or the capacity for prenatal care clinics to address food insecurity among their patients. We aimed to assess barriers and facilitators prenatal care clinics face in addressing food insecurity among pregnant people and to identify opportunities to improve food security among this population.

**Methods:**

We conducted a qualitative study among prenatal care clinics in New Hampshire and Vermont. Staff and clinicians engaged in food security screening and intervention processes at clinics affiliated with the Northern New England Perinatal Quality Improvement Network (NNEPQIN) were recruited to participate in key informant interviews. Thematic analysis was used to identify prominent themes in the interview data.

**Results:**

Nine staff members or clinicians were enrolled and participated in key informant interviews. Key barriers to food security screening and interventions included lack of protocols and dedicated staff at the clinic as well as community factors such as availability of food distribution services and transportation. Facilitators of screening and intervention included a supportive culture at the clinic, trusting relationships between patients and clinicians, and availability of clinic-based and community resources.

**Conclusion:**

Prenatal care settings present an important opportunity to identify and address food insecurity among pregnant people, yet most practices lack specific protocols for screening. Our findings indicate that more systematic processes for screening and referrals, dedicated staff, and onsite food programs that address transportation and other access barriers could improve the capacity of prenatal care clinics to improve food security during pregnancy.

**Supplementary Information:**

The online version contains supplementary material available at 10.1186/s40795-022-00499-7.

## Background

Food insecurity during pregnancy has important implications for the health of the mother and the newborn. Food insecurity during pregnancy has been associated with maternal stress, weight gain, and gestational diabetes [1, 2], low birth weight [3], certain birth defects [4], premature birth and hospitalizations for infants less than 6 months of age [5], and inadequate infant feeding practices [6]. Food insecurity can impact dietary quality during pregnancy, which also contributes to negative maternal health and birth outcomes [7–9]. Due to the central role of maternal nutrition during pregnancy, food insecurity as well as dietary quality can have long term consequences for child growth and development [10].

There is a critical need to understand how to best screen for and address social risk factors—including food, housing, transportation, and other factors [11]—within health care settings generally [12]. This is particularly important in prenatal care settings, given mounting evidence that supports interventions to reduce risk factors such as food insecurity [13]. Screening for food insecurity during prenatal care visits identifies those at-risk and provides an early opportunity for intervention to support the health of mother and child [5]. Health care settings and their staff can help address food needs among patients by implementing evidence-based screening approaches, referring to community-based resources, connecting patients to public nutrition assistance programs, and providing onsite food and nutrition support [14].

Before the COVID-19 pandemic, food insecurity impacted one in 10 households in the United States, with higher rates among rural households and those with children. Among rural households, 12.1% experienced food insecurity in 2019 compared to 10.3% in urban areas. At the state level, 6.6% of New Hampshire households and 9.6% of Vermont households were food insecure in 2019.[15] The COVID-19 pandemic exacerbated food insecurity across the country [16]. Preliminary estimates from New England states showed much higher rates during the pandemic: for example, between 18.7–29.0% in Vermont and 34.1% in Maine [17].

Northern New England has among the highest rates of ‘at least adequate prenatal care’ in the country (90.9% in VT, 87.1% in ME, and 84.9% in NH) [18]. Identifying pregnant people in food insecure households provides a unique opportunity to connect them with the Supplemental Nutrition Assistance Program for Women, Infants, and Children (WIC). Other interventions should be considered as well. For example, food prescription programs can link patients to nutrition support programs either onsite at the clinic or offsite through a community partnership [19]. Group prenatal care that emphasizes nutrition education has been shown to improve food security status compared to individual care [20].

However, there is limited evidence about the implementation of food security screening within prenatal care settings or the capacity for clinics to address food insecurity among their patients. We aimed to assess barriers and facilitators prenatal care clinics face in addressing food insecurity among pregnant people in northern New England. These findings can help to identify opportunities to improve food security among pregnant people in this region.

## Methods

We conducted a qualitative study using key informant interviews among staff and clinicians at prenatal care clinics within the Northern New England Perinatal Quality Improvement Network (NNEPQIN). NNEPQIN is a voluntary consortium of approximately 50 hospitals, outpatient clinics, and other organizations involved in perinatal care in ME, NH, and VT convened to improve the rapid dissemination of evidence-based practices in prenatal, intrapartum, and newborn care. Northern New England is a predominantly rural area with small urban centers throughout.

The qualitative findings reported here are a component of a mixed methods study on food security procedures in NNEPQIN practices. A clinician or staff member engaged in each clinic’s food security work was invited to participate in a brief survey about food security (quantitative data are not presented due to small sample size). Recruitment was conducted via a network wide email and direct email outreach to network members. Survey participants were asked to indicate their interest in participating in a key informant interview and were subsequently invited to complete an interview. Key informant interview participants received a $50 gift card.

A semi-structured interview guide developed by the research team was used to explore barriers and facilitators to screening for and addressing food insecurity among pregnant and postpartum patients. Interviews lasted 30–60 min and were conducted by trained members of the research team over the phone, audio recorded, and transcribed verbatim for analysis.

Qualitative data were analyzed with Dedoose 8.3.47 software [21]. Thematic coding was used to identify relevant excerpts in the data. The research team developed a preliminary codebook of a priori codes based on literature review, content covered in quantitative surveys, and observations from interviews. The codebook was modified, and emergent codes were added based on consensus conversation as coding progressed. Each interview was independently coded by two research team members who then worked in pairs to reach complete agreement on final code application. For the most frequently used codes (used 10 times or more), excerpts were extracted and reviewed to identify major themes. Three researchers reviewed the coded data to identify major themes and through an iterative process came to consensus on final themes. For each thematic area, illustrative excerpts were selected.

This study was reviewed and determined exempt by the Dartmouth-Hitchcock Health System IRB.

## Results

Nine participants from eight distinct clinics completed a semi-structured telephone interview. Participant and clinic characteristics are presented in Table 1. The majority of participants were based in hospital-affiliated clinics and considered food security to be very important. They reported using both formal and informal mechanisms (i.e. through patient dialogue with no formal screening tool) for screening for food security. The most frequently used codes are in Table 2. The most frequently used codes described staff involved in screening for food insecurity, changes in community resources due to COVID-19, improvements in interventions for addressing food needs, acceptability to patients, and onsite and offsite interventions.

**Table 1:** Key informant interview participant characteristics (*n* = 9)

Characteristic	n
**Respondent Type**	
Provider (i.e. physician, nurse practitioner, or physician assistant)	2
Clinical Nurse (RN, LPN)	3
Care coordinator	1
Social worker	2
Resource specialist or community health worker	1
**Practice Type**	
Hospital-affiliated	7
Federally Qualified Health Center (FQHC)	2
**Practice Location**	
Rural	4
Urban	5
**Food Need Importance**	
Very important	7
Somewhat important	2
**Screening Type**	
Formal	4
Informal	5

*SW* social worker, *CHW* Community Health Worker

Rural = Rural–Urban Commuting Area Code ≥ 4

**Table 2:** Most frequently used codes

Code/sub-code	Description	Frequency
**Screening**		
Tool	Which screening tool a practice uses	15
Staff	Staff members and clinicians involved in screening processes	28
Workflow	The steps and workflow for screening	18
Method	How screening is performed (e.g. on paper or in an electronic health record)	14
Improvements	What changes the participant would make to the current screening processes	16
**Intervention**		
Onsite Type	Types of onsite interventions, including internal referrals	22
Offsite Type	Types of offsite interventions, including referrals to external organizations and state-sponsored interventions (e.g. WIC, SNAP)	24
Workflow	The steps and workflow for interventions	13
Improvements	What changes the participant would make to the current interventions	25
**Community**		
Type	Types of food resources available in the community	21
**Patient needs and resources**		
Transportation	Discussions of patient transportation in general	20
Acceptability	Acceptability to patients of discussing/addressing FI in healthcare settings	24
**Practice factors**		
Support needed	What support would be helpful to improve how practices address food insecurity (both screening and interventions)	13
**COVID-19**		
Community	Discussions of changes in coordination with community partners during the pandemic; changes in patient eligibility requirements for food resources at practice or community organizations; new or lost community resources	30
Practice	How the pandemic has changed practice factors (e.g. staff roles or responsibilities, or communication)	13

### Food security screening

#### Screening process

Initial screening for food insecurity was most likely to be carried out by an intake nurse or front office manager using a form that included standard questions on food, nutrition, and other social determinants of health. Some respondents noted that food was part of a general resource screening while others only mentioned screening for food. Intakes were usually completed at the time of the first prenatal care visit.

*“We have a universal prenatal intake process, where someone coming into care for pregnancy, would first have a visit with a registered nurse who fulfills the role of prenatal care coordinator. She does ask questions about... It's a resource security question, I think is how it's phrased like, "Do you have what you need at home?" And then she'll give the examples of, "Do you have shelter? Do you have electricity? Do you have running water? Is it safe? Do you have food to eat?" So it's a question that's along those lines. And then she also asks people about their diet, what they might typically eat in a day. And if they have any restrictions on their diet or things that they avoid.” --Physician A*

There was some variation as to whether screening was standard, i.e. developed externally for use across facilities; clinic-specific, i.e. developed by staff within the clinic; or informal, i.e. motivated staff asking about food or resource insecurity but without consistency. The intake was administered on paper or an electronic tablet, either by a clinician or self-administered. Even if a standard form was used, its implementation could be ad hoc depending on the clinical workload. At times, follow up was done by a prenatal care clinician as a supplement to the initial intake. Several options were mentioned, including follow up by a prenatal care nurse, midwife, or physician in reviewing answers or the ‘problem list’ generated at the initial intake; additional screening and meeting with a social worker; and follow up with community health workers.

#### Barriers to screening

Inconsistency in follow up beyond the intake was noted by several respondents, often attributed to staff workflow and the patient load. The consensus was that it was better to have someone assigned to carry out the initial screening because it was more likely to be completed for every patient, although there were also benefits to having multiple staff/clinicians engaged in the process:

*“It’s helpful to have multiple people who are responsible for asking this because it establishes that as a culture that this is an important part of healthcare.” –Physician B*

Additional follow up or screening was clinic or provider-specific, where some were more proactive than others. Clinic readiness to implement food insecurity screening varied, with some reporting a smoother uptake process than others due to clinic level management and workload:

*“It's been at least 10 years that the clinic has had a prenatal care coordinator, nursing position… And I think it was not difficult to start because it philosophically aligned with the way the clinic is run. It's a very team-based, multidisciplinary clinic, so having a nurse intake coordinator, I don't think, was a heavy lift when they implemented that.” --Physician A*

*“I think that they would be open to hearing about something like that, but I'm not sure that they would want to add something like another assessment onto the already long list of assessments that everyone is responsible for.” --Social Worker A*

Perceived embarrassment and stigma associated with being food insecure, especially for patients who are already parents, was reported as a barrier to screening accurately for food insecurity.

*“I think some of them are not completely honest, you know, because they’re ashamed, or, you know, they’re worried that they can’t provide food for the children that they may have, afraid that we might may call DCYF [Division for Children, Youth and Families] on them.” –Clinical Nurse C*

#### Facilitators to screening

Responses varied with some reporting better outcomes from face-to-face conversations rather than over the phone or on a tablet, especially if other social issues were present. In-person screening was also seen as being more helpful for asking follow up questions about the capacity of the woman or family to access and prepare food.

*“And how are they going to store that food? Are they living with a friend? Are they living out of a hotel? Do they have a refrigerator? I think there's just a lot of assessing that needs to go on in conjunction with food screening. Like, do you have a clean place to prepare the food? Should we be giving it by a food bank? Do you have the means to cook it? They may be living in a hotel and they only have a microwave.” --Clinical Nurse A*

In terms of achieving honest perspectives, allowing for privacy during the intake (either one-on-one with a clinician or self-administered) and giving time to develop a trusting relationship with clinicians were seen as relevant factors for improving communication.

*“We find sometimes, the first visit with the nurse that's their first time here, you're just meeting the person for the first time, it takes a little time to develop a relationship, have them feel comfortable. So they will see myself or the other nurse that works here and then they'll see the social worker and it's a couple weeks later … and then the provider will see all of that information. And then the provider will again ask, but she won't ask everything again. She'll just, if I identity that that woman has domestic violence or has no money for food, does not have resources in place, then she'll follow up again. So we're all trying to get the same information and making sure that the woman feels comfortable talking with us.” --Clinical Nurse B*

Integration of food security with other social risk screening was generally seen as a helpful way to identify women with needs.

*“I do the ones for people that have a substance use history, even if that's just marijuana...so I pop in just to see how they're doing. And those are questions that I always ask, "Do you need diapers? Do you need food? Anything going on with housing?" All those questions are questions that all of us always ask people.” --Social Worker A*

*“We're asking about food. We're also asking about personal safety, depression and housing stability. And to be perfectly honest, I think people are less self-conscious about answering questions about food than they are about the other things” --Social Worker B*

### Areas for improvement in screening

One consistent area of improvement noted by several respondents was more frequent screening throughout pregnancy. Additionally, improvements in screening tools and processes were desired, both for capturing more patients experiencing food insecurity and for ease of use and appropriate referral:

*“If somebody had sort of like a plug and play kind of program and was like, ‘Use this questionnaire, identify these resources and refer to these resources, check in one week, three weeks and 12 weeks or whatever.’ Then I feel like that would be a lot easier than trying to develop it from the ground up because to be honest with you the nurses and the physicians are not trained in this so much.” --Physician B*

*“I think having a very specific screening tool would be helpful, to define what severity is this? Is it a patient not having access to purchasing food, or what level of severity of that? Like, do they know where their next meal is coming from or, do they just not have enough funding to buy healthy food, or they're eating more processed food? I think if we could get into specifically what the food needs are, it would be easier to refer them based off of that.” --Clinical Nurse A*

Respondents also talked about more detailed assessments of food practices and dietary quality to identify specific areas where more targeted interventions may be needed.

*“So it is one of the resources that we give out to patients when they're newly pregnant, is like this is what healthy eating looks like. It's a nice one that you hang up on the wall that has the food group, how much calcium they should be eating for their pregnancy. So it's a great reference to say like, from this food diagram or food pyramid, are you able to eat in all of these tiers? If they're stuck in the process green one, then we need to make a referral so that they can get, and protein and stuff like that.” --Clinical Nurse A*

Clinic staff were hesitant to ask about food insecurity if they were not aware of what interventions were available for their patients. Respondents tied screening for food security to strong interventions that address patient needs once they are identified.

*“But we've noticed that providers are a little more willing to engage with the social needs questions if they have some idea of what the patient is then going to navigate, to be able to get that need met.” --Physician A*

### Interventions for food insecurity

#### Facilitators

The primary means by which clinics addressed a food need was through an internal referral to a clinic-based resource specialist, social worker, or other clinician. Clinics benefited from having a clear referral process in place. In addition, some claimed they were better positioned to implement internal referrals because the clinic placed a greater value on food security as part of health care. For internal referrals to be successful, respondents emphasized the importance of a dedicated resource specialist at the clinic.

*“And if they need to fill out paperwork, she will help guide them and help fill that out with them, which is great, because I think half the time when you try to give a patient resources and make referral, I think the most intimidating part of that is them trying to figure out how to self-navigate through that. And us we can go online and try and figure out what that process is, but having that resource specialist, like she knows what the paperwork is, she knows who the point people are for that resource, and it's just super helpful to have her and know exactly what the process is. And patients are more likely to follow through with that if they have someone helping them through it. Otherwise, they know food banks are out there, but they don't know the 20 steps between knowing that they're there and actually getting food from them.” --Clinical Nurse A*

Respondents also noted a desire to offer onsite food provision services. Providing food directly to pregnant people while at the clinic for an appointment can help to address urgent hunger needs and overcome transportation and accessibility barriers to community resources. Some clinics had services in place to provide food to patients, snacks during appointments, or cafeteria vouchers.

*“I mean I have had people say, we need meat and produce, because that's all we get at the food pantry are non-perishables and canned goods. So that's something that we're fortunate to be able to have milk, and sometimes eggs, and frozen meats, and stuff to give to people because they aren't able to get all that stuff a lot of times.” --Social Worker A*

External referrals to community resources were another means by which clinics addressed food needs among pregnant patients. The most frequently cited resource for pregnant people was the Supplemental Nutrition Assistance Program for Women, Infants and Children (WIC). Clinics relied on easy referrals and strong relationships with WIC to help people access these benefits.

*“Actually, whether a woman identified concerns about food or not, I would always make a referral to WIC, and for food stamps, and facilitate the initial appointments. Let's see. And I got to tell you, that of all the referrals that I made, that was the easiest referral. That was the smoothest referral that I was ever able to make to anybody because the WIC clinic had somebody who would answer the phone, schedule appointments, ask questions, and then follow up. So that was pretty seamless.” --Social Worker B*

#### Barriers

Respondents noted a need for more accessible services in the community, including better hours at local food shelves. Referrals were more effective when there were strong relationships in place between the clinic and the community organization. Respondents also commented on a need for better coordination between clinics and various community resources.

*“We have a ton of community resources and a lot of really well-meaning people and we all have the same goal of supporting these moms. We're trying really hard to get all of these resources together in a way where there isn’t overlap or gaps. And the thing is that some of these resources are independent, some of them are church based, some of them are state supported, some of them are based on grants. If the grant goes away, they go away. Then we've got the nonprofit hospital. And so what we're finding is there's a lot of bandwidth, there's a lot of goodwill. But we wonder about, is there a way that we could more efficiently coordinate all of it?” --Physician B*

#### Patient-level barriers

Transportation was noted as a key barrier that should be addressed when making referrals to community services.

*“I just feel like once you ask about food insecurity, I feel like from there, it will... There may be other needs. ‘Okay, then here's this food pantry.’ And then it's like, ‘Yeah, I understand the food pantry is there, but I don't know how to get there,’ or ‘I don't have internet.’ I feel like there needs to be someone, like a case manager, being able to provide other supports and services as well.” --Care Coordinator A*

Another barrier for patients was lack of awareness about available services. Respondents discussed having lists and information about community resources that could be shared with patients and a dedicated staff member who could maintain relationships with community partners and keep up to date about their services.

*“I think the biggest one is just them not knowing what's out there. Like a lot of them aren't aware that there are food pantries. There's so many like in the community that are near them that they don't even know exists. They don't know that they qualify for WIC or SNAP. So I think it's just like, there's not really a general knowledge of the resources that are out there for them. --Resource Specialist A*

Other barriers were related to communication challenges due to patient stress associated with the experience of food insecurity as well as cultural differences leading to varied understandings of food insecurity between clinicians/staff and patients.

*“I think there's also the psychic challenge of always having to be aware that you have food insecurity. I think that it is depressing and it is exhausting and it is anxiety provoking. And I think that folks get to the point where they just don't want to think and talk about it. And I think that's hard too." --Physician B*

*“The only thing I could think of that could be a barrier is the women that come from a different country. If it's part of their culture not to really share information about that or language barrier, we could be missing some of that with them. It’s hard for me to know if we are if they're not being forthcoming about it.” --Clinical Nurse B*

#### Patient-level facilitators

In general, respondents felt that patients had a relatively high degree of acceptability for discussing food needs with their care team. They noted that patients generally felt comfortable asking for help when they needed it, especially when there was trust between staff and patients.

*“And I think that's where it comes in that my role is important because I'm the connection for them at the clinic. They see me and talk to me on a regular basis, so they're comfortable talking to me. And that goes for a lot of the other case managers too. If it's someone that they see on a regular basis, then that person is comfortable and has an easier time asking for support and knowing what's available. So again, it's the setting of our clinic just kind of lends itself toward that community friendly relationship, I guess.” --Social Worker A*

Commitment at the clinic level and staff buy-in facilitated the process of screening and intervention. Clinics that recognized food insecurity as an important health issue for their patients were better able to develop trusting relationships with patients and address their needs.

*“I actually just really think it's the staff commitment and the team that works here really knows that it's important, nutrition is a very important part of pregnancy and promoting optimal outcomes for pregnancy and health families, so it's really just been a part of our program here since the beginning… It's a very small office… and I think that patients feel that and feel comfortable with us so they will reach out to myself or the social worker and say, ‘I'm really struggling this month, I don't have money enough to get this or this or this.’ So we will put them in the right direction, supplement with that gift card if we have to, but it's really just been part of our clinic and training here.” --Clinical Nurse B*

## Discussion

We aimed to assess barriers and facilitators faced by prenatal care clinics in northern New England in addressing food insecurity among their patients and to identify opportunities to improve food security among this population. Although processes varied between clinics, all participants perceived food security as important for their patients and communities and discussed methods to identify and help patients experiencing food insecurity. The capacity to screen for and intervene on food insecurity was influenced by several barriers and facilitators including whether standard screening tools were used, consistency of screening, availability of food distribution programs in the community, trusting relationships between providers and patients, and the value placed on food security within clinics. Interviews also identified a variety of patient-level barriers such as lack of public and private transportation options and lack of information about community food resource availability, including location and hours of operation.

Only one participant reported the use of a specific food security screening tool during prenatal care visits and the remainder used informal processes that were described as lacking consistency in how food insecurity was assessed, who conducted the screening, and the timing and frequency of screening. Those who used informal screening processes discussed interest in implementing more formal processes. Similar to our findings, previous research has shown that a defined process for screening and referrals during well child care visits can improve access to community resources for families with young infants [22].

Interviewees also described variations in screening and intervention practices tied to the presence or absence of a social worker or resource specialist responsible for addressing social needs. The dedication of at least one key staff member was seen as a facilitator for addressing patient food needs by ensuring screening took place and that patients were appropriately connected to resources.

Participants also described the importance of the clinic’s culture in terms of creating a supportive environment where providers can talk openly about food insecurity and patients feel comfortable discussing their needs and are willing to accept help. A supportive culture has several advantages: encouraging clinicians to follow up on identified patient needs, promoting proactive knowledge of community resources, and establishing partnerships with community organizations, and helping patients to overcome perceived stigma and develop a shared understanding of healthy eating during pregnancy. Communication and trust were seen as important aspects of improving patient acceptability of food assistance.

Relationships between clinics and community organizations were also important, a finding that is aligned with previous qualitative research exploring links between primary care and community organizations for improving food security [23].

### Recommendations

We offer four recommendations to improve the capacity for identifying and addressing food insecurity among pregnant people in prenatal care settings. First, we recommend developing and implementing systematic processes within clinics. To avoid duplication of efforts and share implementation strategies, clinics could be engaged to develop or adapt processes through existing networks. This could include promotion of standard screening tools (e.g. the Hunger Vital sign two item food security screener [24], assessment of dietary quality) and defining workflows for screening and referrals. Workflows should also address the frequency with which screening is conducted.

Second, participants indicated that a dedicated staff person who is responsible for following up on screening results and/or connecting patients with resources facilitates a clinic’s ability to address food insecurity. This role could be filled by a social worker, community health worker, or other resource specialist; however, where resource constraints exist, other existing staff roles could be used. Responsibilities may include carrying out or following up on initial screening, offering onsite food and nutrition support, maintaining relationships with community partners, providing community resources lists, and making referrals. Although a dedicated staff person would be ideal, having multiple provider and staff roles involved in follow up can be helpful to ensure needs are fully met and to help create a supportive environment.

Third, the ability to offer onsite food support within the clinic can motivate care teams to discuss food needs, build trust with patients, address urgent needs, and remove transportation and other barriers to food access. Onsite support could be in the form of a preventive food pantry [25], shelf-stable food boxes, snacks available during appointments, and/or provision of supplemental items such as formula. Having a direct and immediate way to intervene can help engage patients in conversations about food needs, demonstrate the clinic’s commitment and ability to help, and mitigate the stigma associated with seeking outside food assistance.

Finally, developing partnerships with community organizations can facilitate referrals to community resources. Strong partnerships can improve coordination between clinics and longer term food support offered in the community, such as local SNAP and WIC offices, food pantries and other human service organizations. In addition, partnerships can help to raise awareness among both clinicians and patients about available food supports.

### Limitations

There are several limitations of this study. Our sample size was small and comprised of clinics already involved in perinatal quality improvement work (i.e. NNEPQIN members). Therefore, our results may not be generalizable to other geographic regions. In addition, our findings are based on the perspectives of interview participants who may not have complete knowledge of community resources. Despite these limitations, this study is one of the first to specifically address food security within prenatal care settings, and to be conducted in a primarily rural setting. More research on best practices for food security screening and interventions within prenatal care clinics is needed, including research on the experiences of pregnant patients with food security screening and interventions during prenatal care.

## Conclusion

Prenatal care is an opportune setting to identify and address food insecurity among pregnant people. The use of informal processes for food security screening and interventions limits the capacity of the health system to address this important gap in perinatal health. Our findings indicate that more systematic processes for screening and referrals and dedicated staff could improve the ability of prenatal care clinics to address food insecurity.

## Supplementary Information

**Additional file 1.** Semi-Structured Interview Guide

## Data Availability

The datasets generated and analysed during the current study are not publicly available due to privacy and confidentiality reasons but de-identified data are available from the corresponding author on reasonable request.

## Abbreviations

FQHC: Federally Qualified Health Center
NNEPQIN: Northern New England Perinatal Quality Improvement Network
WIC: Supplemental Nutrition Assistance Program for Women, Infants, and Children
