# Supplementary material for: Missed opportunities to improve food security for pregnant people: a qualitative study of prenatal care settings in Northern New England during the COVID-19 pandemic
Source: BMC Nutr. 2022 Jan 24;8:8. doi: 10.1186/s40795-022-00499-7 (PMC8784232; doi:10.1186/s40795-022-00499-7)
Supplement: Supplementary file 1 — Additional file 1. Semi-Structured Interview Guide [file 40795_2022_499_MOESM1_ESM.pdf]

Semi-Structured Interview Guide

These questions will guide the interview but may be adjusted as needed during the interview process.

1. Please describe your role in your clinic.
    a. How long have you been in this position?

Food Insecurity Screening

2. What are your clinic's processes for identifying pregnant women experiencing food insecurity, if any?
    a. How were these processes developed?
    b. How difficult was the process to implement?

If any form of screening exists, 3-4; :

3. What has helped your practice implement and sustain these processes?
    a. What about your clinic environment has helped?
        i. The staff?
        ii. The workflow?
    b. What about your patient population has helped?
    c. Are there any external policies or community factors that have helped?

4. What barriers has your practice faced in implementing food insecurity screening?
    a. Any challenges related to your practice environment?
        i. The staff?
        ii. The workflow?
    b. Any challenges related to your patient population?
    c. Any challenges related to external policies or community factors?

If no formal screening process in place, 5a-b; 6-9:

5. How interested would your practice be in adopting a formal screening process for food security?
    a. What challenges or barriers might prevent your practice from screening?
        i. Any challenges related to your practice environment?
            1. The staff? The workflow?
        ii. Any challenges related to your patient population?
        iii. Any challenges related to external policies or community factors?

    b. What would need to change in order for your practice to adopt a formal screening process for food insecurity?
        i. Change in workflow?
        ii. Change in available resources?

Food Insecurity Interventions

6. What kinds of interventions does your practice offer patients for food insecurity, if any?
    a. Any resources or interventions within the clinic?
    b. Any linkages to community resources outside the clinic?
    c. How has this changed, if at all, during the COVID-19 pandemic?

7. What has helped your practice implement and sustain these processes?
    a. What about your practice environment has helped?
        i. The staff?
        ii. The workflow?
    b. What about your patient population has helped?
    c. Are there any external policies or community factors that have helped?

8. What barriers has your practice faced in implementing food insecurity interventions?
    a. Any challenges related to your practice environment?
        i. The staff?
        ii. The workflow?
    b. Any challenges related to your patient population?
    c. Any challenges related to external policies or community factors?

9. Describe your practice's relationship with community organizations that address food insecurity
    a. Relationships with existing partners?
    b. New partnerships?
    a. Have these practices changed as a result of the COVID-19 pandemic?

Community Resources to Promote Food Security

10. The next few questions are about your patients. You may not know their challenges completely but this will help us to understand if there are resources they could access.
    a. What barriers do patients experience accessing resources to help with food insecurity?
    b. What kinds of community resources are available, if any?
    c. How connected is your practice to the community resources?
    d. Are there any resources that would be helpful but are not available?
    e. *For Rural practices only:* Are there any factors related to your practice's rural setting?
    f. Have needs changed since the COVID-19 pandemic?

Future support

11. What kind of support would help your practice to address food insecurity going forward?
